# Supplementary material for: Multi‐dataset identification of innovative feature genes and molecular mechanisms in keratoconus
Source: J Cell Mol Med. 2024 Sep 19;28(18):e70079. doi: 10.1111/jcmm.70079 (PMC11412914; doi:10.1111/jcmm.70079)
Supplement: Supplementary file 2 — Material S2. [file JCMM-28-e70079-s002.zip › 7.GSEA analysis/5.RNF217-AS1/KEGG_BIOSYNTHESIS_OF_UNSATURATED_FATTY_ACIDS.html]

Details for gene set KEGG\_BIOSYNTHESIS\_OF\_UNSATURATED\_FATTY\_ACIDS[GSEA]

|  || Dataset | RNF217-AS1.group.cls#h\_versus\_l |
| Phenotype | group.cls#h\_versus\_l |
| Upregulated in class | h |
| GeneSet | KEGG\_BIOSYNTHESIS\_OF\_UNSATURATED\_FATTY\_ACIDS |
| Enrichment Score (ES) | 0.16472246 |
| Normalized Enrichment Score (NES) | 0.56873536 |
| Nominal p-value | 0.97987926 |
| FDR q-value | 0.9642733 |
| FWER p-Value | 1.0 |
Table: GSEA Results Summary

  

Fig 1: Enrichment plot: KEGG\_BIOSYNTHESIS\_OF\_UNSATURATED\_FATTY\_ACIDS      
 Profile of the Running ES Score & Positions of GeneSet Members on the Rank Ordered List

  

| SYMBOL | TITLE | RANK IN GENE LIST | RANK METRIC SCORE | RUNNING ES | CORE ENRICHMENT || 1 | BAAT | na | 1973 | 0.352 | 0.1495 | Yes |
| 2 | SCD5 | na | 7187 | 0.147 | 0.0856 | Yes |
| 3 | ACAA1 | na | 7710 | 0.135 | 0.1494 | Yes |
| 4 | HSD17B12 | na | 9226 | 0.101 | 0.1647 | Yes |
| 5 | ACOT7 | na | 14913 | 0.020 | 0.0126 | No |
| 6 | ACOX3 | na | 15596 | 0.013 | 0.0005 | No |
| 7 | ACOX1 | na | 15678 | 0.012 | 0.0053 | No |
| 8 | PECR | na | 16443 | 0.004 | -0.0143 | No |
| 9 | FADS2 | na | 17728 | -0.008 | -0.0464 | No |
| 10 | TECR | na | 19031 | -0.020 | -0.0720 | No |
| 11 | YOD1 | na | 20187 | -0.032 | -0.0867 | No |
| 12 | HADHA | na | 20646 | -0.036 | -0.0786 | No |
| 13 | SCD | na | 21074 | -0.041 | -0.0671 | No |
| 14 | ELOVL6 | na | 22814 | -0.060 | -0.0822 | No |
| 15 | ELOVL5 | na | 24130 | -0.077 | -0.0750 | No |
| 16 | FADS1 | na | 25331 | -0.096 | -0.0534 | No |
| 17 | ACOT1 | na | 27901 | -0.146 | -0.0418 | No |
| 18 | ELOVL2 | na | 28147 | -0.152 | 0.0403 | No |
| 19 | ACOT4 | na | 31309 | -0.255 | 0.0985 | No |
Table: GSEA details [plain text format]

  

Fig 2: KEGG\_BIOSYNTHESIS\_OF\_UNSATURATED\_FATTY\_ACIDS      
 Blue-Pink O' Gram in the Space of the Analyzed GeneSet

  

Fig 3: KEGG\_BIOSYNTHESIS\_OF\_UNSATURATED\_FATTY\_ACIDS: Random ES distribution      
 Gene set null distribution of ES for **KEGG\_BIOSYNTHESIS\_OF\_UNSATURATED\_FATTY\_ACIDS**
